# Investigations on the occurrence of tapeworm infections in German horse populations with comparison of different antibody detection methods based on saliva and serum samples

**DOI:** 10.1186/s13071-020-04318-5

**Published:** 2020-09-10

**Authors:** Laura Jürgenschellert, Jürgen Krücken, Corrine J. Austin, Kirsty L. Lightbody, Eric Bousquet, Georg von Samson-Himmelstjerna

**Affiliations:** 1 Institute for Parasitology and Tropical Veterinary Medicine, Freie Universität Berlin, Robert-von-Ostertag-Str. 7-13, Berlin, 14163 Germany; 2 Austin Davis Biologics Ltd, Unit 1 Denfield Lodge, Lower Street, Great Addington, Northants, NN14 4BL UK; 3 Virbac, 13e rue LID BP 27, Carros cedex, 06511 France

**Keywords:** Equine parasites, *Anoplocephala*, Cestodes, ELISA, Faecal egg count

## Abstract

**Background:**

Effective and sustainable worm control in horses would benefit from detailed information about the current regional occurrence of tapeworms. Different diagnostic methods are currently available to detect *Anoplocephala* spp. infections in horses. However, the format as well as the sensitivity and specificity of the methods vary considerably.

**Methods:**

A coprological, serological and questionnaire study was conducted to investigate the prevalence and risk factors of tapeworm infections on 48 horse farms in the region of Berlin and Brandenburg, Germany. In total, faecal samples of 484 horses were analysed using the double centrifugation/combined sedimentation-flotation and mini-FLOTAC. Serum (*n* = 481) and saliva (*n* = 365) samples were analysed by ELISAs to determine antibody levels against *Anoplocephala* spp. 12/13 kDa excretory/secretory (E/S) antigens.

**Results:**

Cestode eggs were detected in 0.6% of faecal samples (farm prevalence 6.3%) without differences between the two methods. In contrast, antibodies against *Anoplocephala* spp. were detected in 16.2% (farm prevalence 52.1%) and in 29.5% (farm prevalence 75.7%) of the serum and saliva samples, respectively. Both ELISA based methods for detection of tapeworms reported a greater number of infected animals requiring treatment than were positively identified by coproscopy. Logistic regression analysis identified permanent pasture access, large pastures and regular pasture changes and high strongyle egg counts as risk factors for positive serum antibody responses to *Anoplocephala* spp. while last treatment with praziquantel was protective. Other protective factors were the presence of foals and high numbers of horses on the farm. Daily removal of faeces from the pasture and horse age did not have a significant effect.

**Conclusions:**

The findings of the present serological investigation indicate that tapeworm prevalence in Berlin/Brandenburg horse farms is much higher than would be anticipated by using conventional/coproscopic analyses. Moreover, the majority of tapeworm-positive horses had not received a cestocidal drug at their last treatment. Considering the already known low sensitivity of the coproscopic detection, the equine veterinary diagnostics can be enhanced by the use of antibody detection methods such as the saliva-based ELISA. 
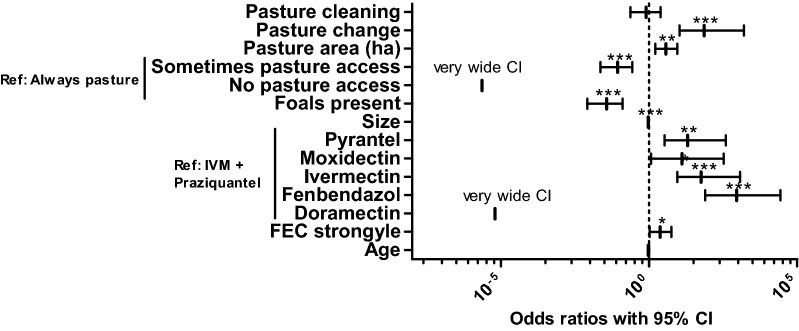

**Electronic supplementary material:**

The online version of this article (doi:10.1186/s13071-020-04318-5) contains supplementary material, which is available to authorized users.

## Background

Horses are particularly prone to infections with ubiquitous gastrointestinal helminth parasites during the grazing period. A major risk factor for *Anoplocephala* spp. is access to pasture [1]. The infestation with tapeworms is caused by the oral uptake of infected oribatid mites (Acari: Oribatida), intermediate hosts carrying the infectious cysticercoid metacestode [2].

Tapeworm species infecting horses as definitive hosts are *Anoplocephala perfoliata*, *Anoplocephala magna* and *Paranoplocephala mamillana* (Eucestoda: Anoplocephalidae) [3]. Here the species are summarised as *Anoplocephala* spp. since *P. mamillana* is considered to be relatively rare and the *Anoplocephala* species are the species of predominant veterinary importance [4]. The prepatency of *A. perfoliata* ranges from one and a half to four months [5]. The detection of cestode prevalence in horses strongly depends on the methods used (Table 1). *Post-mortem* studies conducted in Germany have previously revealed *A. perfoliata* infection in 11.0% to 75.0% of the examined horses [6–9]. Studies conducted using faecal examination of horses determined a 2.0% prevalence of tapeworms in Brandenburg, Germany (farm level 14.3%) [10] and of 3.0% (farm level 35.2%) in northern Germany [11]. Infections with *A. perfoliata* can lead to clinical signs of colic and cause pathological alterations to the intestinal mucosa at the attachment site on the ileocaecal junction and the caecal wall, in particular in the case of horses chronically infected with a large number of worms [4, 12–14]. An infection with *A. perfoliata* is a significant risk factor for ileal impaction colic. This also applies for the occurrence of spasmodic colic in horses, whereas in this case the risk increases with higher worm burden [4].

**Table 1:** Previous studies on equine tapeworm infections in Germany

Region	Year^a^	Study design		Species and prevalence (%)	References
		Method	HL/FL		
Brandenburg	2006	Combined sedimentation-flotation, McMaster	HL	*Anoplocephala* spp. (2.0)	Hinney et al. [10]
			FL	*Anoplocephala* spp. (14.3)	
Lower Saxony	2000, 2001	McMaster (modified)	HL	*Anoplocephala* spp. (0.2)	Wirtherle [39]
Bavaria	2002, 2003	Post mortem sectio	HL	*A. perfoliata* (28.5); *P. mamillana* (1.0)	Rehbein et al. [9]
Bavaria	na	Post mortem sectio	HL	*A. perfoliata* (38.0)	Beelitz and Gothe [6]
		Combined sedimentation-flotation	HL	*A. perfoliata* (18.0)	
Northern Germany	1998, 1999	Combined sedimentation-flotation	HL	*Anoplocephala* spp. (3.0)	Behrens [11]
			FL	*Anoplocephala* spp. (35.2)	
Lower Saxony	1995	Post mortem sectio	HL	*A. perfoliata* (75.0); *A. magna* (12.5); *P. mamillana* (12.5)	Cirak et al. [8]
Nation-wide	1958	Post mortem sectio	HL	*Anoplocephala* spp. (11.0)	Kiedrowski [7]

*Anoplocephala* spp. may include *Anoplocephala perfoliata*, *Anoplocephala magna* and *Paranoplocephala mamillana*

*HL* horse level, *FL* farm level

^a^Sampling year

Cestode eggs are released when the gravid proglottids are detached from the tapeworm and shed in the faeces. Since the eggs are not evenly distributed in faeces, coproscopical analysis is not reliable and suffers from poor sensitivity [12, 15, 16]. Studies conducted using different faecal analysis methods for the detection of cestode eggs rated the semiquantitative combined sedimentation-flotation with concentrated sugar solution for flotation as the most sensitive coproscopical method [17–19]. However, Slocombe [20] reported a sensitivity of 62% and a specificity of 100% using the Cornell-Wisconsin centrifugal flotation technique with 5 g faeces, and a sensitivity of 100% when testing faecal samples 18 hours after anticestodal treatment. Rehbein et al. [18] compared the different results obtained when combined sedimentation-flotation was performed with varying amounts of faeces and type of flotation medium. A total of 74.8% of the sampled horses tested positive when considering the combined results of all methods, while 72.8% of all the horses were found to be positive when using 15 g faeces and saturated saccharose solution as flotation medium. Proudman & Edwards [17] determined a sensitivity of 61% for this method when compared to determination of total worm counts at necropsy. When evaluating sensitivity, the exclusion of false negative results in animals with less than 20 tapeworms led to an increase in sensitivity to 92%. No or weak correlation between infection intensity and the number of cestode eggs per gram of faeces was observed in some surveys [12, 17, 21]. On the other hand, Kjaer et al. [22] reported a significant correlation (Spearman’s rank coefficient of 0.71) and considered the combined sedimentation-flotation using a large amount of sample material (30 g faeces) as a method to detect potentially pathogenic infection intensities (> 20 tapeworms/horse [14]).

The insufficient sensitivity of the coproscopic examinations led to the development of more advanced diagnostic methods. Therefore, serum-based ELISAs [23–25] and a coproantigen ELISA [26, 27] were developed. Recently, a saliva-based ELISA detecting *Anoplocephala*-specific antibodies using a non-invasive approach [28] was conceived as an improved diagnostic option. Other investigations focused on the development of PCR assays to detect and discriminate equine tapeworm stages in faeces [3, 29].

Sensitivities and specificities were not published for all of these assays but the sensitivity of the serum-based ELISA of Proudman & Trees [25] was 68% and the specificity was 95% (in helminth-naive horses). The serum-based *Anoplocephala* ELISA described by Lightbody et al. [28] with 85% sensitivity and 78% specificity is a modified version of the ELISA published by Proudman & Trees [23]. For the saliva-based ELISA [28] 83% sensitivity and 85% specificity were reported. Current immunodiagnostic methods for the detection of 12/13 kDa E/S *A. perfoliata* antigens are probably not sufficiently specific to discriminate between the two *Anoplocephala* species [30] whereas little or no cross-reactivity occurs with *P. mamillana* [23, 25].

The study described here aimed to provide an overview about the current prevalence and risk factors of *Anoplocephala* spp. infections in horses in Brandenburg and Berlin, Germany. Further, the saliva tapeworm ELISA was compared to its serum equivalent in terms of sensitivity as well as to the coproscopic diagnosis.

## Methods

### Study design and location

The study was carried out between May 2017 and January 2018 and included 484 domestic horses of different ages (9 months to 34 years) from 48 horse farms in the federal states Berlin and Brandenburg, Germany. All members on the mailing list of the Berlin-Brandenburg Regional Equestrian Association (LPBB, as of 07.04.2017) were contacted by e-mail. In addition, an advertisement was placed in a regional horse journal and an online ad on the corresponding homepage (www.reiten-zucht.de). This study was made public to other horse owners through social media (https://de-de.facebook.com). Each farm responding to the contact mail or advertisements, holding at least four horses and meeting the requirements regarding the last deworming was included in the study. Horses or farms were excluded if the last anthelmintic treatment of the horses with praziquantel or pyrantel pamoate in the cestocidal dosage of 13.2 mg pyrantel/kg body weight was carried out less than four months prior to sampling. Other anthelmintic treatments during this period did not result in exclusion from the study if no anticestodal effect was to be expected. Serum and faecal samples were taken from each horse. Saliva samples were collected from June 2017 to the end of the study using a saliva collection kit (Austin Davis Biologics Ltd., Northamptonshire, UK). Between four and 17 horses were sampled per farm (aiming at 50 farms and 10 horses on each farm). In this study, the authors define strategic deworming management as a regularly performed anthelmintic treatment of all horses on a farm without prior investigation to identify any parasitic infections that indicate treatment. Selective deworming was defined as an anthelmintic treatment of horses after a given diagnosis, most commonly based on faecal egg counting. A questionnaire was filled out with the farm manager or a horse owner at the day of sampling (Additional file 1: Text S1). Questions were asked about parameters of the farm such as number of animals and presence of foals, and about pasture and hygiene management. Further information on the current deworming management as well as horse parameters such as the age were also collected.

### Coproscopic analyses

Since it was aimed to collect data about *Anoplocephala* and strongyle nematodes, optimised coproscopic analysis methods for both parasite groups were used. Faecal egg counts (FEC) were determined using mini-FLOTAC and a double centrifugation/combined sedimentation-flotation technique in parallel. Prior to the mini-FLOTAC [31], 5 g faecal samples were processed with the Fill-FLOTAC apparatus as described by Noel et al. [32], with 45 ml of saturated saline solution (specific gravity 1.2 (NaCl)) added. For each sample, two 1-ml flotation chambers of the mini-FLOTAC device were counted corresponding to a multiplication factor of 5 to convert raw counts into eggs per gram (epg) of faeces data. The double centrifugation/combined sedimentation-flotation technique was performed as described by Rehbein et al. [18] with slight modifications. For each individual faecal sample 15 g of faeces were utilised. After the first centrifugation, the supernatant was decanted, and the pellet was floated using a concentrated sugar solution (specific gravity 1.26).

### Serum and saliva analyses

Serum samples from 48 farms were analysed with the Horse Serum Tapeworm ELISA (Austin Davis Biologics Ltd, Northamptonshire, UK) [28]. The cut-off values are set as follows: a serum score of < 2.7 is considered to be a negative result; a serum score between 2.7 and 6.3 corresponds to a borderline but positive result; and a serum score of > 6.3 indicates a moderate/high infection, including clinically relevant tapeworm burdens of more than 20 tapeworms [28]. Up to 9 ml of blood were collected from each horse in sterile polypropylene tubes. On the same day, the blood was centrifuged at 2000×*g* for 10 min and the serum was aliquoted into sterile tubes. Samples were stored at −20 °C and shipped on dry ice between laboratories.

Saliva samples from 37 horse farms were collected and tested using the EquiSal® Tapeworm Saliva Test (Austin Davis Biologics Ltd., Northamptonshire, UK) [28]. The resulting saliva score leads to the diagnosis of low (< −0.09), borderline but positive (0.09–0.6) or moderate/high (> 0.6) tapeworm burden. Following saliva collection, the swabs were placed into the preservative solution provided in the test kit and stored at 4 °C. Samples were shipped without cooling within ten days after sampling and placed at 4 °C until testing was carried out. Samples are stable for at least three weeks at room temperature once in the preservative solution (unpublished data). Both *Anoplocephala* assays were performed within three weeks after sampling in the laboratories of Austin Davis Biologics Ltd. (Northamptonshire, UK).

### Data analyses

Data were entered into a Microsoft Excel spreadsheet. Correlation analysis and all graphs were created using GraphPad Prism® Version 5.03. All other statistical analyses were performed with R 3.4.4 using RStudio version 1.1.456 for Windows. Confidence intervals for proportions were calculated as Wilson Score intervals with finite population correction in OpenEpi Version 3.01 [33]. A mid-p exact test for differences between proportions was conducted using the tab2by2.test() function from the *epitools* package version 0.5-10. A logistic regression analysis was performed to identify variables with potential influence on the odds of a horse in the study population to be positive for antibodies against *Anoplocephala* spp. in the serum-based tapeworm ELISA. The serum-based tapeworm ELISA was chosen here due to the fact that the saliva ELISA was not available in the beginning of the study and thus the dataset was smaller. Logistic regression models were calculated using the glm() function in R. The final logistic regression model for explanatory variables that probably affect the odds of a horse being tested positive in *Anoplocephala* spp. serum ELISA was fitted using stepwise backwards elimination with the drop1() function aiming to minimise the Akaike information criterion (AIC). The data for the variable treatment schedule were arranged in four categories. A distinction was made between selective and strategic deworming and the latter were graded according to annual treatment frequencies in low (1–2 treatments per year), moderate (3 treatments per year) and high (> 4 treatments per year). The variables limited and unlimited pasture access were defined according to whether the horses had hourly access to the pasture or unlimited access all day long during the warmer period of the year. To evaluate the logistic regression model, different pseudo-*R*^2^ values were calculated with the PseudoR2() function from the *DescTools 0.99.27* package.

## Results

### Study population

Table 2 provides general data of the study population. The majority of horse owners employed a strategic deworming management with an anthelmintic treatment schedule of 2 to 4 times a year. In this study, 42 horses from five farms received an anthelmintic treatment only irregularly or on suspicion of parasite infection caused by equine gastrointestinal nematodes or cestodes. Among them was only one horse farm that dewormed based on indication due to previous faecal analyses. This also applied to one horse that had recently been placed on a strategically deworming farm. One farm manager stated that deworming was only carried out in cases of suspected endoparasitic disease when the horses were in a reduced general condition. Two farm managers stated that they irregularly sent faecal samples for coproscopic diagnosis. No coproscopic diagnosis was performed on any of the other horse farms. Only 7.2% of the horses had received a treatment containing praziquantel as last anthelmintic therapy. A combination of ivermectin and praziquantel was administered to each of these horses. Praziquantel alone or in combination with moxidectin were not used for any horse in the study population at the last anthelmintic treatment.

**Table 2:** General data of 484 horses from 48 farms included in the study

Parameter	Value
Age (years; median range)	12.0 (0.8–34.0)
Mares (%)	50.2
Geldings (%)	46.7
Stallions (%)	3.1
Faecal samples (%)	100
Saliva samples (%)	75.4
Serum samples (%)	99.4
Last treatment	
Ivermectin + Praziquantel (%)	7.2
Ivermectin (%)	42.4
Moxidectin (%)	4.1
Doramectin (%)	1.4
Fenbendazole (%)	4.3
Pyrantel (%)	38.2
Unknown	2.3
Period between last anthelmintic treatment and sampling (weeks; median range)	14.4 (1.4–100.0)
Horses sampled per farm (number; median, range)	10 (4–17)
Pasture access (%)	96.7
Unlimited (%)	28.9
Limited (%)	67.8
Pasture area/horse (ha) (mean, range)	0.6 (0.1–2.0)
Size/number of horses per farm (mean, range)	40.2 (6–110)
Foals present (%)	39.6
Treatment schedule (annual)	
Selective (%)	8.7
Low number (1–2) of treatments (%)	32.8
Moderate number (3) of treatments (%)	39.6
High number (≥4) treatments (%)	18.9

### Faecal examination

Altogether, 484 faecal samples were examined coproscopically. The estimated prevalence of *Anoplocephala* spp. is presented in Table 3 for the different methods. Eggs of *Anoplocephala* spp. were found in 3 faecal samples (0.6%, 95% CI 0.2–1.8%). The farm prevalence of detected *Anoplocephala* spp. eggs in faeces was 6.3% (95% CI 2.1–16.8%). All three samples were tested positive with both mini-FLOTAC and combined sedimentation-flotation and originated from different farms.

**Table 3:** Prevalence of *Anoplocephala* spp. using different methods

Method	Prevalence (%)	95% confidence interval (%)	*n*/*N*
Sedimentation-flotation	0.6	0.2–1.8	3/484
Mini-FLOTAC	0.6	0.2–1.8	3/484
Serum-ELISA	16.2	13.2–19.8	78/481
Saliva-ELISA	29.5	25.1–34.5	108/365

*n* number in the category *N* total number

In addition, strongyle eggs were observed in 66.7% (95% CI 62.4–70.8%), *Parascaris* spp. in 0.4% (95% CI 0.01–1.5%) and *Oxyuris equi* in 1.2% (95% CI 0.6–2.7%) of the samples. These figures refer to the combined results of both faecal analysis methods. None of the samples was positive for *Strongyloides westeri* or *Habronema muscae*.

### Serum ELISA

Of the 481 collected serum samples, 16.2% (95% CI 13.2–19.8%) (Table 3) tested positive in the serum-based tapeworm ELISA and were diagnosed as borderline (9.1%; 95% CI 6.9–12.1%) or moderate/high (7.1%; 95% CI 5.5–9.7%). On the farm level, 52.1% (95% CI 38.3–65.5%) were positive with at least one horse on the farm reporting a borderline serum score with antibodies against *Anoplocephala* spp.

### Saliva ELISA

Between June 2017 and January 2018, 365 saliva samples from 37 horse farms were tested with the saliva-based ELISA. Altogether 29.5% (95% CI 25.1–34.5%) of the tested horses were diagnosed positive (Table 3) including samples with borderline (9.6%; 95% CI 7.0–13.0) or moderate/high (20.0%; 95% CI 16.2–24.4) saliva scores. On 75.7% (95% CI 59.9–86.6%) of the farms at least one horse was diagnosed to be positive (both borderline and moderate/high) by the saliva-based tapeworm ELISA. Table 4 provides details of the determined farm prevalence using the two ELISA tests.

**Table 4:** Prevalence in serum- and saliva-based ELISA for all 48 farms

Farm number	Total no. of horses on farm	*N*	Prevalence in serum (95% CI) (%)	*N*	Prevalence in saliva (95% CI) (%)
1	32	10	40.0 (20.9–68.7)	na	na
2	21	10	0 (0.0–27.8)	na	na
3	80	10	0 (0.0–27.8)	na	na
4	38	10	0 (0.0–27.8)	na	na
5	85	10	10.0 (2.9–39.4)	na	na
6^a^	30	10	50.0 (28.1–71.9)	na	na
7	44	10	10.0 (3.9–38.3)	na	na
8	29	10	0 (0.0–27.8)	na	na
9	50	10	0 (0.0–27.8)	na	na
10	25	10	50.0 (29.2–70.8)	na	na
11	14	12	0 (0.0–24.3)	na	na
12	60	10	0 (0.0–27.8)	10	20.0 (7.5–49.2)
13	30	10	0 (0.0–27.8)	10	0 (0.0–27.8)
14	95	10	0 (0.0–27.8)	10	0 (0.0–27.8)
15	75	12	0 (0.0–24.3)	11	27.3 (11.4–54.9)
16	18	10	0 (0.0–27.8)	10	10.0 (7.9–34.4)
17	44	10	40.0 (19.7–65.9)	10	60.0 (34.1–80.3)
18	22	8	0 (0.0–32.4)	8	25.0 (11.9–54.3)
19	110	10	0 (0.0–27.8)	9	33.3 (13.0–63.6)
20	90	10	30.0 (12.1–59.0)	10	50.0 (25.0–75.0)
21	18	12	25.0 (23.4–51.4)	12	75.0 (55.8–82.1)
22	23	10	10.0 (6.3–36.0)	10	0 (0.0–27.8)
23	90	9	22.2 (7.4–53.6)	9	33.3 (13.4–63.4)
24	35	10	60.0 (35.0–79.5)	10	80.0 (52.3–91.1)
25	11	10	40.0 (34.6–51.0)	10	40.0 (34.6–51.0)
26	22	10	40.0 (34.6–51.0)	10	80.0 (54.5–88.8)
27	85	5	0 (0.0-43.5)	5	20.0 (4.3–61.7)
28^a^	12	10	60.0 (46.2–68.3)	10	100 (80.2–100.0)
29	35	10	20.0 (8.9–47.8)	8	0 (0.0–32.4)
30	33	11	0 (0.0–25.9)	11	0 (0.0–25.9)
31	12	8	0 (0.0–32.4)	8	25.0 (17.5–48.8)
32	25	5	0 (0.0–43.5)	7	57.1 (29.1–80.2)
33	35	10	10.0 (4.5–37.7)	10	10.0 (4.5–37.7)
34	20	10	0 (0.0–27.8)	10	0 (0.0–27.8)
35	25	16	18.8 (13.7–40.0)	16	31.3 (22.9–47.6)
36	100	15	0 (0.0–27.8)	13	15.4 (5.5–41.1)
37	20	10	0 (0.0–27.8)	10	0 (0.0–27.8)
38	28	12	33.3 (19.2–55.5)	12	50.0 (31.1–69.0)
39	26	10	20.0 (10.2–46.5)	10	20.0 (10.2–46.5)
40 ^a^	37	8	100 (67.6–100.0)	8	87.5 (55.2–95.5)
41	15	11	9.1 (1.0–29.3)	11	36.4 (26.7–53.1)
42	15	8	12.5 (8.8–40.5)	8	37.5 (21.9–61.3)
43	35	16	0 (0.0–19.4)	16	6.3 (4.5–24.9)
44	20	9	0 (0.0–29.9)	9	0 (0.0–29.9)
45	40	8	37.5 (16.3–66.8)	8	37.5 (16.3–66.8)
46	29	17	17.6 (12.2–35.0)	17	23.5 (16.1–40.8)
47	6	4	25.0 (16.6–57.9)	4	25.0 (16.6–57.9)
48	85	5	0 (0.0–43.5)	5	0 (0.0–43.5)

^a^Farm with a horse tested *Anoplocephala* spp. positive in the faecal analysis

*CI* confidence interval, *na* not available

### Comparison between coproscopic, serum and saliva testing

Altogether, the results of 363 matching samples from the saliva, serum and faecal analysis were obtained. Each of the three horses, which were *Anoplocephala* spp. positive in the faecal analysis, had a moderate/high score in the serum ELISA. From one of these three horses, no saliva sample was collected; the other two had saliva scores indicating a moderate/high tapeworm burden. Considering the prevalence in serum- and saliva-based ELISA on the farms (Table 4), it appears that the horses that were *Anoplocephala* spp. positive in faecal analysis were found on farms with a high prevalence.

**Fig. 1:**
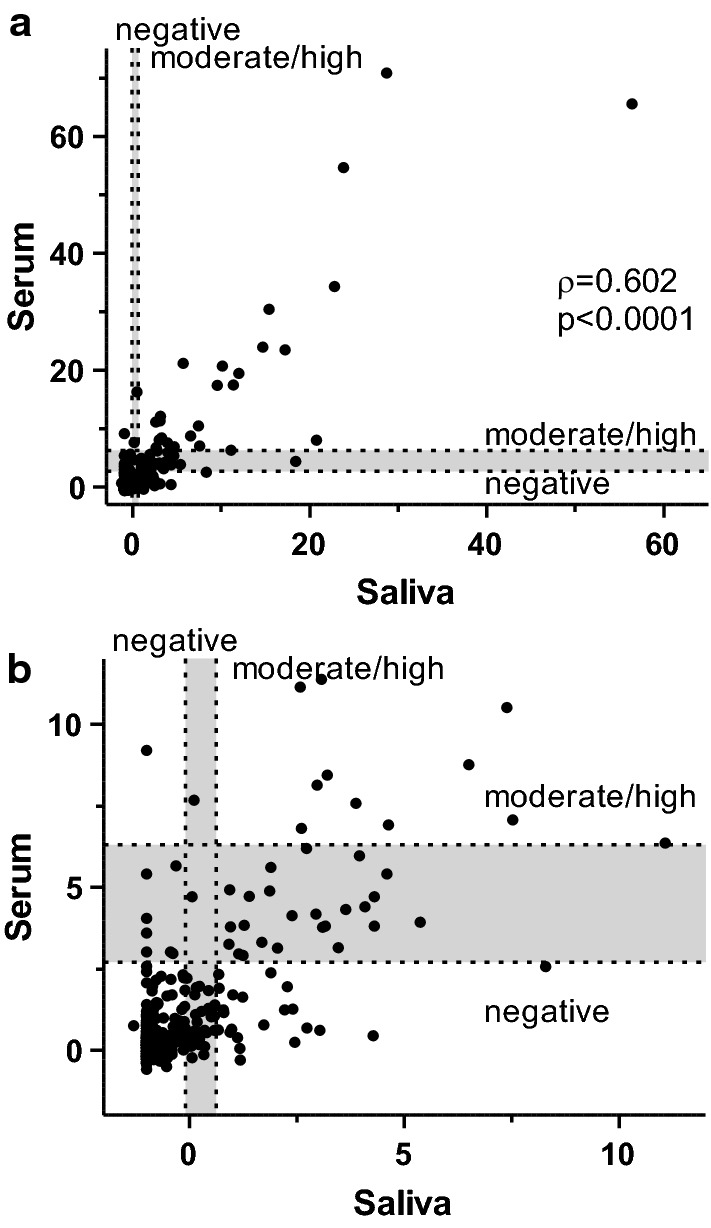
Comparison of the serum and saliva scores for the detection of antibodies against *Anoplocephala* spp. Serum scores were plotted over saliva scores for 363 horse samples for which both datasets were available. **a** Complete dataset. **b** Enlarged view of the lower left area of (**a**) with 16 data points out of axis limitations. Cut-off values are indicated by spotted horizontal and vertical lines. Serum tapeworm ELISA: cut-off: < 2.7 low; > 6.3 moderate/high. Saliva-based ELISA: Cut-off: < −0.09 low; > 0.62 moderate/high. Positive but borderline score areas are indicated by grey shading

Among the 363 matching samples, 106 (29.2%, 95% CI 24.8–34.1) were positive in the saliva-based ELISA and 61 (16.8%, 95% CI 13.3–21.0%) in the serum-based ELISA. This difference was statistically significantly different in the mid-p exact test (*P* < 0.0001). The scores calculated for the paired serum and saliva ELISAs are plotted against each other in Fig. 1. Since data were not normally distributed, Spearmanʼs correlation index was calculated. There was a highly significant correlation of serum and saliva scores (*P* < 0.0001) and the Spearman correlation coefficient of *ρ* = 0.602 indicated a moderate positive correlation [34].

Inter-rater agreement was evaluated using kappa (κ) statistics. There was a significant agreement between both tests (*P* < 0.05) and the unweighted Cohen’s κ coefficient to measure the inter-rater agreement of saliva-based and serum-based ELISA for the detection of antibodies directed against *Anoplocephala* spp. resulted in a value of 0.54 (95% CI 0.44–0.63), which indicates a considerable agreement [35]. For 16.7% of the matching samples, saliva and serum assays produced disagreeing results but in the majority of these cases (62.3%) the positive assay reported a borderline score, i.e. above the cut-off for negative samples but below the cut-off for moderate/high tapeworm burdens (Fig. 1).

### Risk factor analysis

**Fig. 2:**
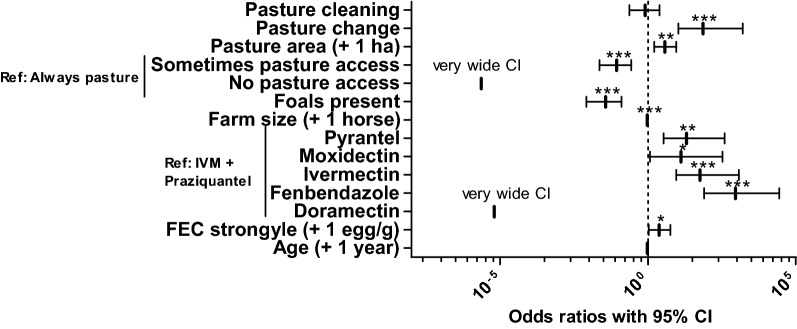
Forest plot showing odds ratios (with 95% confidence intervals) for the final logistic regression model for explanatory variable potentially influencing the odds to be positive in the *Anoplocephala* serum ELISA. For the bivariate variables pasture cleaning (daily removal of faeces), pasture change (regular change between different pastures) and foals present (at least one foal on farm) the reference level is “no”. For multilevel variables, the reference level is given in the figure. Metric variables included are the pasture area (in ha/horse), number of horses on the farm, faecal egg counts (FEC) of strongyle nematodes, and age of the horse (in years). The variable pasture access was divided in the levels: always pasture, i.e. permanent access to pasture during the grazing season (reference category); sometimes pasture access (hourly access to the pasture during grazing season); and no pasture access (no pasture access at all). For the variable last anthelmintic treatment, the only level including the highly cestocidal drug praziquantel, which was always given in combination with ivermectin (IVM), was chosen as reference. *P < 0.05, **P < 0.01, ***P < 0.001

The odds ratios of different explanatory variables included in the final model are plotted in Fig. 2. More details on the model are provided in Table 5. The variables treatment schedule and period between the last antiparasitic treatment and sampling were eliminated from the final model. Limited pasture access was protective in comparison with permanent pasture access during grazing season. No pasture access was even more protective, but this was not statistically significant presumably since the number of horses in this category was very small and therefore 95% CIs were very wide. Other pasture related variables in the final model were daily pasture cleaning (no significant effect), the size of the pasture per horse and if horses were regularly moved between pastures. The latter two both were highly significantly associated with higher odds to be infected. The use of praziquantel in the last deworming was significantly associated with lower *Anoplocephala* infection risks compared to all other drugs used for treatment including pyrantel in the dosage recommended for treatment against nematodes. The only exception was doramectin, but here again a very wide 95% CI was encountered since only a very small number of horses all from the same farm represented this dataset. Presence of foals on the farm was associated with low odds to detect *Anoplocephala* antibodies. Very small but significant protective effects were associated with the number of horses on the farm. A small protective effect of the age was not significant but was included in the final model since it decreased its AIC. The high pseudo-R^2^ values according to McFadden & Nagelkerke show that the model is a considerable improvement in comparison to the null model.

**Table 5:** Final logistic regression model to identify risk factors explaining positive samples in *Anoplocephala* serum ELISA

Reference level	Term	Estimate	SE	*P*-value	Odds ratio	Lower 95% CI	Upper 95% CI
	Pasture cleaning	− 0.219	0.590	0.711	0.804	0.236	2.459
	Pasture change	4.288	1.188	< 0.0001	72.836	10.669	1586.703
	Pasture area (+ 1 ha)	1.317	0.438	0.003	3.732	1.613	9.096
Always pasture access	Sometimes pasture access	− 2.443	0.627	< 0.0001	0.087	0.023	0.274
	No pasture access	− 12.971	1475.0	0.993	2.327×10^−6^	8.047×10^−230^	2.976×10^19^
	Foals present	− 3.305	0.692	< 0.0001	0.037	0.008	0.127
	No. of horses (+ 1 horse)	− 0.053	0.014	< 0.0001	0.948	0.921	0.971
Ivermectin + Praziquantel	Pyrantel	2.989	1.108	0.007	19.870	3.363	390.831
	Moxidectin	2.562	1.344	0.057	12.960	1.167	330.938
	Ivermectin	4.048	1.143	< 0.0001	57.261	9.013	1176.149
	Fenbendazole	6.812	1.420	< 0.0001	908.693	79.217	27177.460
	Doramectin	− 11.975	1726.7	0.994	6.300×10^6^	na	3.445×10^149^
	FEC strongyle (+1 egg/g)	0.862	0.428	0.044	2.369	1.061	5.751
	Age (+ 1 year)	− 0.043	0.024	0.075	0.958	0.913	1.003
	(Intercept)	− 5.600	1.697	0.001	0.004	0.000	0.072

Pseudo- $$R_{{_{\text{McFadden}} }}^{2}$$: 0.41; Pseudo-$$R_{{_{\text{Nagelkerke}} }}^{2}$$: 0.54

## Discussion

Cestode eggs were identified in only 0.6% of the coproscopical examinations (farm level 6.3%). Compared to other studies conducted in Germany using faecal sampling (Table 1), this is slightly below the expected frequency in the study population. A prevalence of 2.0% (farm level 14.3%) was observed by Hinney et al. [10] and 3.0% (farm level 35.2%) by Behrens [11]. In the present survey, the three *Anoplocephala* spp. positive faecal samples were detected in both mini-FLOTAC and combined sedimentation-flotation. The double centrifugation/combined sedimentation-flotation technique is recommended for the coproscopical detection of *Anoplocephal*a spp. eggs being relatively sensitive compared to flotation methods [17, 18]. It is noteworthy that herein, no differences were found between the data obtained by the two faecal analysis methods. However, due to the extremely small number of positive samples, this does not allow to draw any general conclusions, e.g. concerning sensitivity or specificity of the two methods.

In contrast to the coproscopically obtained prevalence, the high seroprevalence observed here is in line with the prevalences as determined in previous surveys at abattoirs in the region of Bavaria, Germany (28.5–38.0%) [6, 9]. Recently, Lightbody et al. [36] used the saliva-based ELISA in a longitudinal study with naturally infected horses from the UK and 15% initially tested positive and this value remained approximately constant in follow up visits after six and 12 months [36].

In a direct comparison of the results of the serum and saliva ELISA, a Spearman correlation coefficient of 0.602 was obtained indicating a moderate positive correlation [34]. During the validation of both tests, a considerably higher Spearman rank correlation of 0.87 was observed [28]. In the present study, the Cohenʼs kappa coefficient of 0.54 further indicated a considerable agreement [35] between the data obtained using the serum-based and the saliva-based ELISA. The saliva-based ELISA test ranked 29.5% of saliva samples above the treatment threshold, whereas in serum-based ELISA this applied to 16.2% of the horses tested. Since the sensitivities for both tests as specified by Lightbody et al. [28] with 83% for saliva and 85% for serum are comparable, this difference is considerable. Disagreeing results were particularly often identified in weakly positive samples. Low parasite numbers might be an underlying reason for this observation as previously reported by Lightbody et al. [28].

Proudman & Trees [23] have investigated the decrease of IgG(T) in serum of horses after anticestodal treatment. Although the onset of IgG decay is fast after treatment, it takes several weeks to months for the antibody levels to return to a level below the cut-off for infected horses. Wilson et al. [37] determined a serum half-life of IgG(T) of approximately 35 days. The reduction of tapeworm specific IgG(T) was monitored during validation of the saliva-based ELISA [28]. The kinetics of antibody reduction in saliva was tested after deworming in eleven horses. After 6 weeks, the specific-antibody levels were reduced to below the treatment threshold for all horses [28]. The relatively rapid decrease in saliva antibody levels after treatment suggests that the majority of horses that tested positive in the present study were actually or at least recently infected at the sampling time point. Serum and saliva-based ELISAs indicate the potential for diagnostics on individual horse level. The extent to which this could reduce the use of anticestodal treatments on a regular basis should be further investigated.

The logistic regression analysis identified several risk factors that are obvious and easy to understand. For instance, the protective effect of praziquantel in the last anthelmintic treatment is expected, in particular when considering the short half-live of the antibodies against *Anoplocephala* spp. [28, 37]. Last treatment with the macrocyclic lactones ivermectin or moxidectin alone or the benzimidazole fenbendazole was associated with a higher chance to be seropositive for *Anoplocephala*. In contrast, treatment with doramectin, which was used only on a few horses from the same farm and which is actually not licensed for use in horses, was associated with a very low odds ratio but a very wide confidence interval. The authors do not recommend or support anthelmintic treatment with drugs that are not registered for horses. Pyrantel is licenced for treatment of *Anoplocephala* infections when used at a (double) dose of 13.2 mg pyrantel per kg body weight. Based on previous findings, partial effects even of 6.6 mg/kg pyrantel on *Anoplocephala* for the 185 horses that had received pyrantel as last anthelmintic drug could have been expected [38]. However, in our analysis the last anthelmintic treatment with pyrantel was not associated with lower odds for *Anoplocephala* infections.

Of course, access to pasture, which also means access to oribatid mites as intermediate hosts, must be considered a risk factor for exposure to *Anoplocephala*. Interestingly, in this context pasture hygiene in terms of pasture cleaning had no significant effect and surprisingly changing pastures over the season and a large pasture area per horse came out as risk factors instead of being protective as one could expect since low host densities should also result in low parasite prevalence. Most likely, these variables represent confounders that do not directly but indirectly affect the odds to be positive for anti-*Anoplocephala* antibodies. One possible explanation could be that large pastures and pasture rotation lead to more vegetation on the grassland. This might be beneficial for the oribatid mite populations. Therefore, it could lead to a higher proportion of grass in the total feed of horses that is potentially contaminated with mites. In contrast, many animals staying all over the year on the same pasture could lead to sparse vegetation and supplementation of feed with larger amounts of hay. Thus, large pasture areas might essentially have the same effect as unlimited access to pasture. However, many other factors can be considered, including soil and vegetation type.

Furthermore, the logistic regression showed that odds to be positive for antibodies against *Anoplocephala* were significantly increased with increasing epg for strongyle nematodes. Despite the various differences in the life-cycles of these parasite groups, both are transmitted by grazing and access to grass and low pasture hygiene might influence both in the same direction.

Other variables that showed a significant correlation with the odds to be seropositive for *Anoplocephala* were the presence of foals on the farm and the number of horses per farm. Both effects were highly significant but the effect of the number of horses was only very small (odds ratio 0.95). The effect of presence of foals was considerably larger (odds ratio 0.04). Both effects are difficult to explain, but might be due to different farm management practices. The age of the foals cannot explain the effect since the study population included only one foal.

## Conclusions

In this study, only a few horses were found positive for *Anoplocephala* spp. by faecal analysis. The saliva-based ELISA, as a non-invasive method, detected significantly more horses with a tapeworm antibody titre than with the serum ELISA. The most important protective factor concerning *Anoplocephala* infection was treatment with praziquantel while pasture access was the most prominent risk factor.

## Supplementary information

**Additional file 1: Text S1.** Questionnaire on sampled farms and deworming management.

## Data Availability

The datasets used and analysed during the current study are available from the corresponding author on reasonable request.
