# Supplementary material for: Investigations on the occurrence of tapeworm infections in German horse populations with comparison of different antibody detection methods based on saliva and serum samples
Source: Parasit Vectors. 2020 Sep 10;13:462. doi: 10.1186/s13071-020-04318-5 (PMC7488081; doi:10.1186/s13071-020-04318-5)
Supplement: Supplementary file 1 — Additional file 1: Text S1. Questionnaire on sampled farms and deworming management. [file 13071_2020_4318_MOESM1_ESM.docx]

**Additional file 1: Text S1**

**Survey for the prevalence study on the occurrence of intestinal helminths in horse populations in Berlin and Brandenburg**

This study aims to investigate the occurrence of intestinal helminths in horse farms in Berlin and Brandenburg. Serum, saliva and faecal samples will be collected. Participation in this study is on a voluntary basis. Your data will be treated confidentially and used for research purposes only. The survey is used for the statistical evaluation of the prevalence study within the context of a dissertation at the Department of Veterinary Medicine at the Freie Universität Berlin. The experimental project has been reported to and confirmed by the local authorities (Landesamt für Gesundheit und Soziales Berlin, LAGeSo, approval number Reg 0059/17)).

# Ⅰ. Horse keeping

1. Farm type:

Riding stable (less than 2 foals, more than 15 horses)

Small farm (less than 2 foals, less than 15 horses)

Stud farm (more than 2 foals)

1. Total number of horses:
2. Age of horses: Quantity

Foals (under 1 year):

Yearlings (1-3 years):

Adults (over 3 years):

1. Horse accomodation:

Open stable

Box

Group Box

other

# Ⅱ. Pasture management

1. Do the horses have access to the pasture? yes no

How often?

year around

only in summer

full day

a few hours

1. How many hectares of pasture are used per horse?
2. Are pastures regularly changed?  yes no

If yes, at what intervals?

After anthelmintic treatment?  yes no

1. Do you feed fresh (chopped) grass?  yes no

# Ⅲ. Hygiene – Stable and pasture

1. How often do you clean the stables?
2. Do you disinfect your stable annually?  yes no
3. If yes, what disinfectant is used?
4. Is cleaning or disinfection carried out after deworming?

yes no

1. Do you collect faeces from the pastures?  yes no

How often?

1. Do you use hay racks? yes no

# Ⅳ. Anthelmintic management

1. Are horses dewormed at regular intervals? yes no

Do all horses receive anthelmintic treatment at the same time? yes no

Date of the last anthelmintic treatment:

Which drug was used?

foals:

yearlings:

adults:

breeding mares:

1. How often is anthelmintic treatment given?:
2. Do you change the drug classes regularly?

yes no

How often?

1. Are new horses dewormed before arrival at the farm? yes no
2. Do you follow an evidence-based selective deworming approach based on faecal egg counting?

yes no

Which faecal egg count is the limit above which the horses are treated?

1. Do you monitor the success of the anthelmintic treatment using faecal samples?

yes no

1. Please describe the anthelmintic management:

Many thanks
